# Iowa Medical Department

**Published:** 1853-10

**Authors:** 


### IOWA MEDICAL DEPARTMENT.

Numerous and desirable improvements have recently been made upon and within the College Buildings, contributing both to their elegance, convenience and utility. A thorough and appropriate painting, both within and without, has entirely changed the appearance of the structure. The Museum has been remodeled and much improved.—Among other important additions, we are pleased to state that more than four hundred elegant and richly colored botanical plates, originally from France, have been contributed to the Museum by Prof. Hughes, by whom they were obtained at very considerable expense.

A large collection of casts and a variety of surgical appliances are also contributed by him. A valuable addition to the useful collection of dry preparations was also made to the Museum, in accordance with the expressed directions of the late estimable Dr. Richards, of Dubuque, whose loss has been so deeply felt and so widely lamented. Aside from the great intrinsic value of his last contribution, it is a permanent memento of the interest he felt in the progress of our Institution, of which he had been a Professor, and for which he never ceased to feel and express the warmest attachment.

In addition to the improvements alluded to, desirable changes are also being made in the Hospital, connected with the Medical College. These numerous changes—for a long time needed—are but an outward type of the improved condition and enlarged sphere of usefulness of the Institution. We are happy to be able to announce to the numerous friends of the Medical College, throughout the State, that the position and prospect of the Institution were never as favorable as now. It is already apparent that a more than usually large class of students will attend the course of Lectures during the ensuing winter. At this present writing, more than a month before the Lectures commence, the number of individual applications already received to attend the next course, is much greater than the whole number of students at the last session. We are pleased to be able to make this announcement, because it indicates the increased prosperity of the Medical Department—because it is well calculated to strengthen our hands and stimulate to renewed effort—because it is most gratifying to the honest pride the Faculty feels in the Institution itself and in the future of medical intelligence throughout the State, which the College represents.

*Surgical Notice.*—As it is the desire of the Faculty of this Institution that the citizens of the State should receive as much benefit as can flow from it, and also that the class during the coming winter may be benefitted as much as possible, we would announce to the profession throughout the State, that all surgical operations performed before the class will be *gratuitous.* The Prof. of Surgery has made every arrangement, both in connection with the Hospital and private boarding houses, for the accommodation of all cases that may present themselves.

Prof. H. has returned from the East with ever appliance necessary in his department. Besides instruments for every form of operation, he has procured of Mr. Gemrig of Philadelphia, a full set of apparatus for disease and deformity of the spine or loss of motion in the extremities; also for club foot, and almost every variety of deformity.—He also has instruments and apparatus for all operations upon the eye, as well as a number of artificial eyes of every color and of perfect construction.

